# Enrichment of Anaerobic Microbial Communities from Midgut and Hindgut of Sun Beetle Larvae (*Pachnoda marginata*) on Wheat Straw: Effect of Inoculum Preparation

**DOI:** 10.3390/microorganisms10040761

**Published:** 2022-03-31

**Authors:** Bruna Grosch Schroeder, Washington Logroño, Ulisses Nunes da Rocha, Hauke Harms, Marcell Nikolausz

**Affiliations:** Department of Environmental Microbiology, Helmholtz Centre for Environmental Research—UFZ, 04318 Leipzig, Germany; bruna.schroeder@ufz.de (B.G.S.); washington.logrono@ufz.de (W.L.); ulisses.rocha@ufz.de (U.N.d.R.); hauke.harms@ufz.de (H.H.)

**Keywords:** enrichment, larva gut, lignocellulosic biomass, anaerobic digestion, methane, carboxylates

## Abstract

The *Pachnoda marginata* larva have complex gut microbiota capable of the effective conversion of lignocellulosic biomass. Biotechnological utilization of these microorganisms in an engineered system can be achieved by establishing enrichment cultures using a lignocellulosic substrate. We established enrichment cultures from contents of the midgut and hindgut of the beetle larva using wheat straw in an alkaline medium at mesophilic conditions. Two different inoculation preparations were used: procedure 1 (P1) was performed in a sterile bench under oxic conditions using 0.4% inoculum and small gauge needles. Procedure 2 (P2) was carried out under anoxic conditions using more inoculum (4%) and bigger gauge needles. Higher methane production was achieved with P2, while the highest acetic acid concentrations were observed with P1. In the enrichment cultures, the most abundant bacterial families were Dysgonomonadaceae, Heliobacteriaceae, Ruminococcaceae, and Marinilabiliaceae. Further, the most abundant methanogenic genera were *Methanobrevibacter*, *Methanoculleus,* and *Methanosarcina*. Our observations suggest that in samples processed with P1, the volatile fatty acids were not completely converted to methane. This is supported by the finding that enrichment cultures obtained with P2 included acetoclastic methanogens, which might have prevented the accumulation of acetic acid. We conclude that differences in the inoculum preparation may have a major influence on the outcome of enrichment cultures from the *P. marginata* larvae gut.

## 1. Introduction

According to a recent analysis, the number of studies related to the conversion of waste biomass has grown in recent years [1,2]. The production of biofuels and platform chemicals from agricultural wastes is a promising technology for replacing fossil fuels and fossil-based chemicals from renewable resources that does not compete with feed and food production [2,3]. The biorefinery concept is gaining attention since lignocellulosic biomass can be converted into several products in a cascade process [4,5]. Furthermore, waste biomass is a cheap and widely available raw material [6,7].

Agricultural waste biomass types, such as wheat, corn and rice straw, and sugarcane bagasse, among others, are composed basically of lignin, cellulose, and hemicellulose, serving as structural materials in plants [1]. They vary widely in composition from plant to plant. In particular, the amount of lignin is highly variable and often substantial, thus exerting a major influence on anaerobic processing. Lignin is one of the biggest obstacles to the biological conversion of lignocellulosic biomass. In addition to its recalcitrant nature, it wraps cellulose and hemicellulose, therefore preventing their bioconversion [8]. Cereal straw, deciduous wood, and coniferous wood contain 12–20 wt %, 14–25 wt %, and 23–38 wt % of lignin, respectively [6,9]. Wheat straw is composed by 35–45% cellulose, 20–30% hemicellulose, and 8–15% lignin [10,11,12]. The ratios of these components directly influence the efficiency of the applied bioprocess and the selection of the most appropriate one.

One important process used for converting lignocellulosic biomass into biofuel is anaerobic digestion (AD) to methane-rich biogas. This process consists of four stages: hydrolysis, acidogenesis, acetogenesis, and methanogenesis [13]. Each step relies on a specialized set of microorganisms working simultaneously to produce biogas and other compounds under anaerobic conditions [14]. The process of anaerobic digestion also occurs naturally in various types of environments: in hydromorphic soils; in the sediments at the bottom of water bodies, such as rivers, lakes [15], oceans, swamps, and ponds; in the guts of various insects [16,17,18,19,20,21]; and in the rumen of ruminant animals [22,23]. The gut system of herbivorous insects is more effective in lignocellulosic biomass degradation than traditional biogas reactors [24]. Usually, beetle larvae can feed on a wide range of organic matter [25,26], but lignocellulose is generally a nutrient-poor and fiber-rich diet [27]. Nevertheless, beetle larvae survive on a diet restricted to cellulose. Werner [28] fed and sustained Rose beetle larvae with filter paper for 6 months. Despite the diet being poor in nutrients, the microorganisms present in the larvae’s gut can use cellulose as an exclusive carbon source for the production of essential acids for the survival of the host.

The Scarab beetle (*Pachnoda* sp.) larva gut is considered as a small bioreactor, and its microbiota has been studied [16,17,29,30]. According to Cazemier et al. [30], up to 65% of the fibers ingested by scarab beetle larvae are digested after passing through their gut system. These insects can efficiently digest lignocellulosic biomass and overcome the recalcitrance of this biomass [24]. The *Pachnoda marginata* larva gut is composed of three compartments: the foregut, midgut and hindgut (also called paunch). The foregut has evolved to effectively break down the organic particles ingested by the larvae, working as a mechanical pretreatment of all ingested biomass. The foregut is composed of mandibles, and a proventriculus region with tooth-like structures and a strong muscular layer around them [4]. The midgut of the *Pachnoda* sp. larva is known to have a high pH, around 10–12 [16,29]. The number of (hemi) cellulolytic bacteria and related enzyme activity in this gut section have been reported to be lower than in the hindgut [16,30]. However, the high pH in this region supports the pre-cellulolytic phase by softening the lignocellulosic biomass [30]. The hindgut has a lower pH (between 7 and 8) than the midgut [29]. It is known to harbor 100- to 1000-times more bacteria than the midgut [31]. It was hypothesized that the hindgut bacteria play a key role in the conversion of cellulose and hemicellulose [4,30]. Therefore, all three gut compartments acting synchronously and harmoniously can process organic matter, and convert it into acids and other compounds essential for the larvae’s survival. Millions of years of co-evolution of host and gut microorganisms were necessary for developing and improving these natural systems [4]. The pretreatment (both mechanical and chemical) and processing of organic matter by microorganisms are essential for the success of the sun beetle larvae in nature. Therefore, an analogous process chain may improve the digestion of lignocellulose to carboxylates and methane in engineered systems.

The characteristics of the beetle larva gut make this insect and its gut microbiome an attractive object of study and a potential source of inoculum for engineered systems. However, direct inoculation of even laboratory-scale reactors with larva gut content is not possible due to the size differences. Thus, enrichment technology must be applied as an intermittent step to upscale the inoculum and pre-select those microbiota members best suited for the conditions in the reactor.

Enrichment from different sources, such as Scarabaeidae larvae [4,32,33], cow and goat rumen [22,23], termite gut [20] and soda lakes [15], has been used to establish inocula for engineered systems. However, none of the studies that we reviewed during the preparation of the present study emphasized the methodology of inoculum preparation related to the desired function, for example, the desired product spectrum. In this study, we aimed to enrich microbial communities from *Pachnoda marginata* larvae gut on wheat straw and alkaline culture medium using two different procedures for inoculum preparation. First, we followed a standard sample preparation procedure using lower inoculum volume in an ordinary sterile bench considering the survival and further enrichment of oxygen resistant anaerobes. The second procedure followed a strict anoxic preparation method in an anaerobic glove box using larger inoculum volumes to promote the enrichment of strict anaerobes. Process parameters were analyzed, and differences in the emerging microbial community structures were followed by amplicon sequencing targeting bacteria and methanogenic archaea using 16S rRNA and *mcr*A genes as molecular markers, respectively.

## 2. Materials and Methods

### 2.1. Medium and Wheat Straw Batch Cultivation

Modified DSMZ medium 1036 adjusted to pH 9.0 was used for the whole experiment. The base medium was composed of 2.0 g NaCl, 0.5 g NH_4_Cl, 0.2 g KH_2_PO_4_, 0.2 g KCl, 0.2 g yeast extract, 0.1 g MgCl_2_ × 6 H_2_O, 0.5 mg resazurin, and 1.0 mL trace element solution SL10 (DSMZ medium 320), dissolved in 850 mL high-purity water. The base medium was supplemented with 100 mL NaHCO_3_ (76 g L^−1^), 34 mL Na_2_CO_3_ (29.41 g L^−1^), 12 mL cysteine-HCl monohydrate (30 g L^−1^), and 4 mL selenite-tungstate (DSMZ medium 385, 1:4 diluted with high-purity water) [15]. The pH was adjusted to 9 using NaOH (2 M). Detailed anoxic stock solution and media preparation have been described previously [34,35].

The wheat straw used as the substrate was collected in Saxony, Germany. The wheat straw was ground with SM 2000 equipment (Retsch, Haan, Germany) to obtain particles of 6 mm length. Then, 0.5 g of wheat straw and 1 mL of high-purity water were added to a 100 mL serum bottle. To avoid potential contamination with spore-forming bacteria from the straw substrate, the serum bottles were closed with aluminum foil and autoclaved at 121 °C for 20 min. Afterward, the serum bottles were transferred to the anoxic chamber and left there overnight. The following day, 1 mL of anoxic high-purity water was added to each serum bottle, and closed properly with sterile butyl rubber stoppers and sealed with aluminum caps. Then, they were autoclaved for a second time under the same conditions. Culture bottles were filled with either 49 mL (P1) or 48 mL (P2) of modified DSMZ medium 1036. In both procedures, 50 mL of medium was added to the negative control (NC) bottles.

### 2.2. Inoculum Preparation

*Pachnoda marginata* larvae were obtained from Bugs International GmbH, Irsingen/Unterfeld, Germany, a commercial breeder. The larvae were kept in a plastic box with fresh soil while the beetles were fed with fresh bananas. Prior to their dissection, larvae were numbed in N_2_/CO_2_, 80%/20% ratio for 15 min.

The inoculum preparation was first conducted based on previous experiences in our laboratory (unpublished) and the knowledge that the gut is not strictly anoxic due to the oxygen diffusion through the epithelial tissue [36]. The first inoculation experiment was performed under open-air conditions despite the risk of oxygen inhibition to strict anaerobes (bacteria and methanogens), hereinafter referred as inoculum preparation procedure 1 (P1). Considering the potential of oxygen as an inhibitory factor, we tested whether the inoculum preparation under anoxic conditions could lead to a better methanogenic process, hereinafter referred as inoculum preparation procedure 2 (P2). This approach was considered because previous experiences in our laboratory preparing inoculum under strict anoxic conditions for anaerobic processes obtained satisfactory results [34,35].

For P1, four larvae were dissected in a laminar flow chamber. The midguts were separated from the hindguts and added separately to a 20 mL glass vial containing 5 mL of sterile DSMZ 1036 culture medium. The guts were macerated with sterile glass bars, and the suspensions were subsequently vortexed for 30 s. Afterward, the headspace of the vials was flushed with N2 for 2 min. Then, 1 mL of the inoculum was removed with a large gauge needle and added to each of the serum bottles previously prepared with 49 mL DSMZ 1036 culture medium and wheat straw as described above. Cultures were incubated statically at 37 °C for 30 days. After 30 days of cultivation, a new transfer was performed. First, 0.2 mL (0.4%, *v*/*v*) of liquid culture was removed from the serum bottle with a Ø 0.55 × 25 mm needle, and transferred to new serum bottles with wheat straw and DSMZ 1036 culture medium. Four transfers were performed.

For P2, five larvae were dissected in a laminar flow chamber. The midguts were separated from the hindguts and added separately to a 20 mL glass vial containing 5 mL of sterile DSMZ 1036 culture medium. The vials were transferred to the anoxic chamber for further inoculum preparation. For each compartment of the larva gut, the following procedures were performed: The guts were macerated with metal tweezers. Afterward, a metallic sieve (500 microns) was used to separate the intestinal tissue from the rest of the gut content. Another 5 mL of culture medium was used to remove the residue from the sieve. Then, 1 mL of the inoculum was removed with a large gauge needle and added to serum bottles previously prepared with DSMZ 1036 culture medium and wheat straw as described above. After 30 days of cultivation at 37 °C, a new transfer was performed. First, 2 mL (4%, *v*/*v*) of the culture were removed from the serum bottle with a Ø 0.80 × 40 mm needle, and transferred to new serum bottles with wheat straw and DSMZ 1036 culture medium. Six transfers were performed.

In P1, all triplicate cultures were transferred to new serum bottles after 30 days of incubation. However, gas production was followed for a longer period in most cases (50 days). In P2, only the bottle with the highest methane production among the triplicates was chosen for the new transfer. The enrichment was carried out in triplicate in addition to a negative control. Before each culture transfer, the selected bottles were shaken by swirling, and a well-homogenized liquid sample was used to inoculate the new transfer for both cultivation procedures.

### 2.3. Analytical Methods

Every 5 days, the gas and liquid phases of the serum bottles were sampled. After sampling 1 mL of liquid from each enrichment culture bottle, 100 µL were used to measure pH using a mini-pH meter (ISFET pH meter S2K922, ISFETCOM Co., Ltd., Hidaka, Japan), and 900 µL were collected in a 1.5-mL test tube and centrifuged at 4 °C and 20,817× *g* for 10 min. The supernatant was filtered through a membrane filter with a 0.2 µm pore size (13 mm; LABSOLUTE, Th. Geyer GmbH, Hamburg, Germany). The supernatant was used for measuring the volatile fatty acids using high-performance liquid chromatography (HPLC; Shimadzu Scientific Instruments, Maryland, CO, USA) equipped with a refractive index detector (RID) L-2490 and an ICSep column COREGEL87H3 (Transgenomic Inc., Omaha, NE, USA), as described in detail by Logroño et al. [34]. The pellets were stored at −20 °C until DNA extraction.

Every 5 days, the relative pressure and temperature in the bottles were measured with a digital manometer (Keller LEO5 4 bar, KELLER AG, Winterthur, Switzerland), followed by the sampling of 1 mL of gas and injection into a 20 mL pre-flushed argon vial, as previously described [34]. The gas samples were measured via gas chromatography (Perkin Elmer GC equipped with HayeSep N/Mole Sieve 13X columns and a thermal conductivity detector), as described in detail by Logroño et al. [34]. Using Equation (1), the gas amount in the bottles was calculated.

(1) $$V_{biogas}=\frac{\Delta P\times V_{headspace}\times C}{R\times T}$$

where *V_biogas_* is the volume of produced biogas (mL), ΔP is the difference of measured pressure (kPa), *V_headspace_* refers to the volume of the headspace, *C* is the molar volume of ideal gas (22.41 L mol^−1^), *R* is the universal gas constant (8.314 × 10^4^ mbar cm^3^ mol^−1^ K^−1^), and *T* is the standard temperature in Kelvin.

Using Equation (2), the volume of methane was calculated.

(2) $$V_{CH_{4}}=\left(\frac{CN_{CH_{4}}}{100}\right)\times V_{biogas}$$

where *V_CH_4__* is the volume of methane (mL), *CN_CH_4__* is the normalized methane concentration (%—mol/mol), and *V_biogas_* is the volume of produced biogas (mL).

Using Equation (3), the specific methane production was calculated.

(3) $${C_{spec.}}_{{}_{CH_{4}}}=\left(\frac{V_{CH_{4}}}{m_{\begin{array}{c} wheat\\ straw \end{array}}\times VS_{\begin{array}{c} wheat\\ straw \end{array}}}\right)÷100$$

where ${C_{spec.}}_{{}_{CH_{4}}}$ is the specific methane production per gram volatile solids (mL CH_4_ gVS^−1^), $V_{CH_{4}}$ is the volume of methane (mL), *m_wheat straw_* is the wheat straw mass (g), and *VS_wheat straw_* is the wheat straw volatile solids (%).

After measuring pressures and temperatures, and taking gas and liquid samples, all bottles were degassed until 0.009 bars and placed back in the incubator until the next measurement.

### 2.4. Microbial Community Analysis

The frozen pellets were thawed, and the DNA was extracted with the NucleoSpin^®^Soil Kit (MACHEREY-NAGEL GmbH & Co. KG, Düren, Germany) using SL2 buffer and SX enhancer. The quantity and quality of the extracted DNA were assessed with a NanoDrop spectral photometer (Thermo Fisher Scientific, Waltham, MA, USA) and gel electrophoresis using 0.8% agarose gel and ethidium bromide staining.

The bacterial and methanogenic archaeal communities were analyzed by the amplicon sequencing (Illumina MiSeq) of 16S rRNA and *mcrA* genes, respectively. To amplify the V3–V4 region of 16S rRNA genes, two primers were used, 341f (5′-CCT ACG GGN GGC WGC AG-3′) and 785r (5′-GAC TAC HVG GGT ATC TAA KCC-3′), as described by Klindworth et al. [37]. The PhiX Control v3 Library was used as a control according to the protocol of Illumina. 

The primers used for amplifying *mcrA* genes were the mlas (5′-GGT GGT GTM GGD TTC ACM CAR TA-3′) and mcrA-rev (5′-CGT TCA TBG CGT AGT TVG GRT AGT-3′), as described by Steinberg and Regan [38]. Cutadapt was used for removing primer sequences from adapter-clipped reads. The DADA2 workflow was used for further sequence analysis [39]. Both forward and reverse sequences were truncated to 270 bp by default, which resulted in sequence quality above the Phred quality score of 20 (99% base call accuracy). The amplicon sequence variants (ASVs) obtained from 16S rRNA gene amplicon sequencing were taxonomically classified using the SILVA database [40], and *mcrA* amplicon sequencing was taxonomically classified using the same approach and sequence database as described by Popp et al. [41]. Microbial diversity analyses were performed using the R package “phyloseq” [42]. Bray–Curtis dissimilarity indices and non-metric multi-dimensional scaling (NMDS) plots were built as described by Lian et al. [43] but rarefied to 53,276 reads per sample for 16S rRNA data and 2423 reads per sample for *mcrA* data. The “adonis2” function in “vegan” R package was used for calculating the permutational multivariate analysis of variance (PERMANOVA) [44], applying 106 permutations. Demultiplexed raw sequence data were deposited in the NCBI SRA public database under the bioproject PRJNA788342. ASVs can be used to differentiate treatments with accuracy, and these ASVs can be termed biondicators [45]. In our study, ASVs which could be used to classify the difference between the two inoculum preparation procedures were identified as bioindicators following the three-step process described previously [43,45]. Shortly, we defined bioindicators ASVs that could determine the class (group of treatments) of the different enrichment cultures. The classes were determined using beta-diversity analysis (Bray-Curtis distance among the samples and statistic difference between classes determined by PERMANOVA). We determined the ASVs that most influenced the class separation using Random Forest. First, we tested statistically significant differences in the ASV relative abundances using the LSMEANS test with pair-wise methods adjusted by false discovery rate (FDR) correction. ASVs showing no statistical difference among classes were removed from the analysis. After, we selected the most relevant ASVs using their Mean Decrease Gini indexes. Once these ASVs (bioindicators) were selected, we used them to calculate their error rates to separate the classes determined by beta-diversity analysis using a Random Forest classifier.

## 3. Results and Discussion

### 3.1. Physical-Chemical Characterization of the Inoculum and Enrichment Cultures

**Figure 1:**
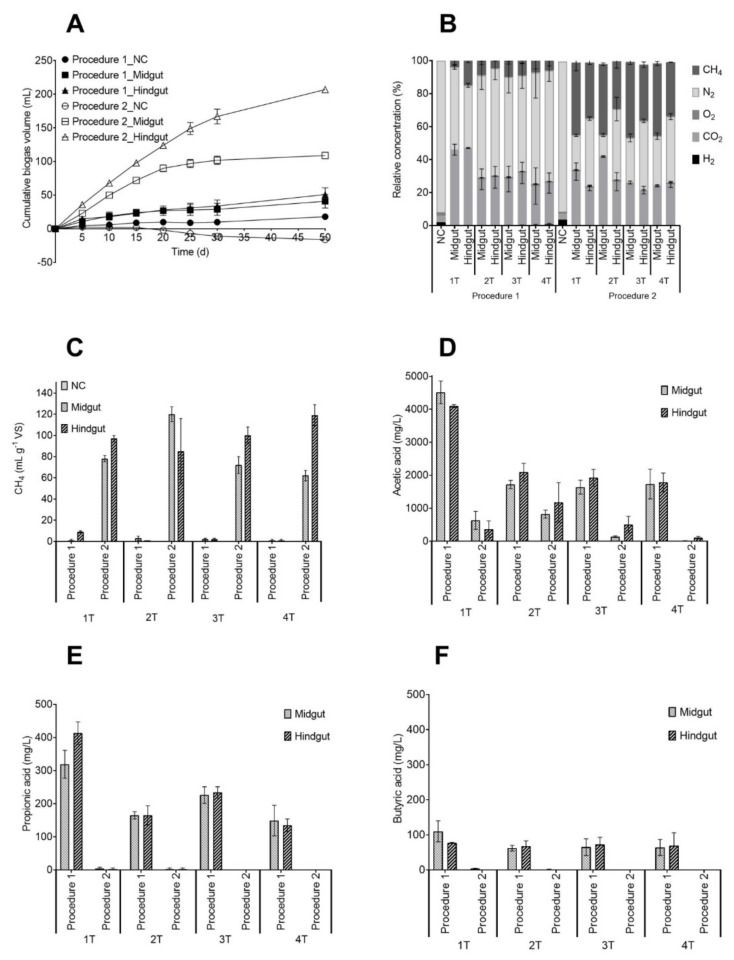
Physiological data of the enrichment cultures from the midgut and hindgut larva compartments using two different inoculum preparation procedures. (**A**) Cumulative gas over 50 days of cultivation in midgut and hindgut cultures during the fourth transfer (4T). (**B**) Relative gas concentration at 30 days of cultivation. (**C**) Methane yield at 30 days of cultivation. (**D**–**F**) Volatile fatty acids concentration at 30 days of cultivation. (**D**) Acetic acid, (**E**) propionic acid, (**F**) butyric acid concentrations. The error bars represent the standard deviation of the mean of n = 3 (invisible error bars are smaller than the symbol). Filled symbols: procedure 1. Open symbols: procedure 2. NC stands for negative controls.

Lignocellulosic biomass-degrading microbial communities were enriched at alkaline pH (9.0) from the midgut and hindgut of the *Pachnoda marginata* larvae, and wheat straw was used as a sole complex carbon and energy source. During this study, two different inoculation preparation procedures were used. P1 (i) was performed entirely in a laminar flow chamber, (ii) 0.4% (*m*/*v*) inoculum was transferred to the new cultivation bottles, (iii) a small gauge needle (Ø 0.55 × 25 mm) was used to inoculate the new cultivation bottles. In the case of P2, (i) the larvae dissection was performed in a laminar flow chamber and the rest of the procedure was performed inside an anaerobic glove box, (ii) 4% (*v*/*v*) inoculum was transferred to the new cultivation bottles, (iii) a bigger gauge needle (Ø 0.80 × 40 mm) was used to inoculate the new cultivation bottles and to transfer bigger solid particles with attached microorganisms. Different amounts of biogas and carboxylates were produced according to the procedure used for inoculum preparation. In general, an accumulation of carboxylates and low biogas production was observed with P1, while the opposite trend, low carboxylate and high biogas production, was observed with P2. In the case of P1, in the fourth transfer (4T), the cumulative gas production after 30 days of cultivation was 29 mL and 34 mL in the midgut and the hindgut cultures, respectively. In the case of P2, the cumulative gas production was 102 mL and 167 mL in the midgut and hindgut cultures, respectively (Figure 1A). These results indicate that preparing the inoculum under anoxic conditions improved biogas and methane production, likely by avoiding the inhibition of strict anaerobes. Compared to previous studies with straw enrichment cultures derived from various sources [23,46], the newly derived P2 enrichment cultures showed a comparable or better performance. The best result of the previous studies was obtained with sheep rumen enrichment culture with a maximum cumulative gas production of 115 mL, which was calculated for the same amount of straw used in this study [46]. Those enrichment cultures were successfully used in bioaugmentation experiments to enhance biogas production [46,47].

The gas composition also varied between inoculation preparations. Methane concentrations were higher for P2 independent of the gut section the inoculum was obtained. Methane concentrations varied from 4% to 15% and from 29% to 46% in the case of P1 and P2, respectively. The CO_2_ concentration did not vary much among the enrichment cultures. For P1, the CO_2_ concentration ranged from 24% to 47%, while for P2, it ranged from 21% to 42% (Figure 1B). The methane yield for P1 was only around 2 mL g_VS_^−1^ in the midgut and hindgut cultures (3T), while for P2, it reached 62 mL g_VS_^−1^ and 119 mL g_VS_^−1^ in midgut and hindgut cultures (3T), respectively (Figure 1C). Consequently, the methane production in the case of P2 was significantly higher for hindgut cultures than midgut, with the exception of the second transfer, where the opposite was observed. Interestingly, the hindgut enrichment culture produced more biogas from straw than previous soda lake-derived mesophilic alkaline enrichment cultures [15] even though similar substrate, cultivation, and medium conditions were applied. Other studies have also found methane production in *Pachnoda marginata* [30], *P. ephippiata* larvae [29], and other taxa of the Scarabaeidae family [48,49]. When the gut sections were analyzed separately, methane was detected only in the hindgut [29], which is in agreement with our study since the highest methane yield was observed in the hindgut enrichment culture. The larvae were kept in closed containers, and re-inoculation of the gut from feces-contaminated soil could explain the successful enrichment of methanogens even from the midgut, which is not considered an environment favorable for the methanogens.

The volatile fatty acid (VFA) concentrations also varied between inoculation procedures. The main product was acetic acid, and the highest concentration for P1 occurred at 30 days of cultivation in the first inoculation, reaching 4515 mg L^−1^ and 4100 mg L^−1^ in midgut and hindgut enrichment cultures, respectively. In the case of P2, the highest acetic acid concentrations were measured at 10 days of cultivation, but after that, the acetic acid was further converted to methane. At 30 days, just before the new transfer, almost no acetic acid was detected in midgut and hindgut enrichment cultures (Figure 1D). Propionic acid was detected in very low concentrations at 30 days of cultivation in P2 and butyric acid was not detected, whereas in P1, the propionic acid concentration in the first transfer was 319 mg L^−1^ and 413 mg L^−1^ for midgut and hindgut cultures, respectively. After four transfers, the concentration decreased to 149 mg L^−1^ and 135 mg L^−1^ for midgut and hindgut cultures, respectively (Figure 1E). Butyric acid was detected at low concentrations, around 65 ±5 mg L^−1^ in all transfers for P1 (Figure 1F), and there was no statistical difference in propionic acid and butyric acid concentrations between the midgut and hindgut cultures (*p* < 0.05). The same was observed for acetic acid concentrations, with an exception for the first transfer (T1). 

Enrichment techniques have been widely used for the discovery and isolation of new microorganisms with desired abilities for given possible biotechnological applications. However, some factors are important concerning the enrichment, especially regarding the inoculum: the inoculum source [50,51], the inoculum age [52,53], the inoculum/substrate ratio [54,55,56,57], the preparation [58,59], and the new artificial conditions to which the inoculum will be submitted. Aeration is a well-known pretreatment method for the preparation of inocula for dark fermentation, as reviewed previously [60]. However, inoculum aeration has been investigated only by a few studies with waste-activated sludge as the inocula, and the results were ambiguous. In addition, the effect of the enrichment process was not investigated in these previous studies by state-of-the-art molecular techniques.

In this study, using two different inoculum preparation procedures, the enriched microbiota of the midgut and hindgut of *P. marginata* larvae were driven toward the production of either biogas or carboxylates. Previous studies that have used the gut microbiome of Sacarabaeidae larvae as inoculum did not mention whether the dissection and inoculum preparation were carried out in an oxygen-free environment [17,29,32,61,62,63,64,65,66,67]. Only one study mentioned that the dissection was carried out in a sterile environment [68]. Differences have been found in the ratio of microorganisms that are associated with solid particles or living freely both in artificial cultures and in the animal guts [59,69,70,71,72]. This suggests that making consecutive culture transfers including not only the transfer of the liquid fraction, but also of solid particles (wheat straw), is crucial for the integrity of the enrichment culture, and the conditions are closer to the real ones found in the larva’s gut. The sterilization of wheat straw was necessary to kill microorganisms present in wheat straw to avoid competition [73]. However, straw sterilization acts as a pretreatment, softening the plant fibers, making cellulose and hemicellulose more available for hydrolysis, and consequently increasing the production of VFAs and biogas [74]. Therefore, it is necessary to consider that an increase in the production of biogas or carboxylates compared to industrial applications using raw straw may be associated not only with the physico-chemical conditions applied during the enrichment process, but also with the pre-stage of wheat straw sterilization.

Degradation of lignocellulosic biomass by microbiota enriched from insect larvae gut, rumen, or soil has been reported in the literature. However, the various types of biomasses, pretreatments, and cultivation conditions make a comparison among these works difficult. Sheng et al. [62] reached 83% degradation of rice straw pretreated with alkaline solution for 3 days using the enriched microbiota of the hindgut of *Holotrichia parallela* affiliated to the Scarabaeidae family. Large amounts of VFAs, mainly acetic acid, have been found in the guts of members of the Scarabaeidae family [48]. In the study of enrichment of the microbiota of four different species of termites, Auer et al. [20] observed that the accumulation of VFA, predominantly acetate, ranged from 2.2 to 5.8 g L^−1^. 

Lazuka et al. [73] achieved 42% wheat straw degradation with enriched termite gut microbiota, observing VFA as the main product. Feng et al. [75] achieved 51% degradation of corn stover powder using enrichment cultures from soils collected from woodlands, but they obtained low VFA production. Thus, the use of insect gut microbiota is equally [16] or more efficient [33] for the production of VFAs than the microbiota of cow rumen that are conventionally used in the anaerobic digestion of materials rich in lignocellulose.

The pH showed the same dynamic in procedures 1 and 2 over 50 days of cultivation in all transfers. Usually, on the fifth day of cultivation, the pH dropped from 9 to 7.7. The pH was slightly lower in procedure 2 for hindgut cultures, where it dropped to pH 7.1 at 50 days of cultivation (data not shown). The pH in the *Pachnoda* sp. larva gut varies between the gut compartments. In the foregut, it ranges from pH 4.1 to 6.7 [16]; in the midgut, from 9.5 to 11.7; and in the hindgut, from 5.7 to 8.3 [16,29]. Even though in this experiment, the initial pH of cultures was set to 9.0, after 5 days of cultivation, the pH had already dropped due to the VFA production. Importantly, the pH of the cultures was not adjusted after the inoculation of the new transfer. The alkaline pH of the larvae’s midgut increases the dissolution of lignin and decreases crystallinity of the biomass [67]. Therefore, it appears to act as a natural pretreatment of the organic matter ingested by the larva, and also acts to facilitate nutrient absorption by the host [76,77,78,79]. Despite the high pH in the midgut of Scarabaeidae larvae, the high enzyme activity in this gut section is not affected [80,81,82]. Thus, the high pH in the midgut of Scarabaeidae larvae seems to be an important adaptation [83]. Alkaline pretreatment studies of wheat straw have resulted in greater lignin removal than other types of pretreatments [84]. Consequently, alkaline pretreatment appears as a good approach for application in anaerobic systems, since in natural systems, lignin can be degraded efficiently only in the presence of oxygen. Unfortunately, it is still a great biotechnological challenge to perfectly mimic the physicochemical conditions of the larvae’s gut in artificial systems and achieve the same lignocellulosic biomass conversion [85], which could lead to an increase in the either production of carboxylates or biogas.

### 3.2. Bacterial Community Structure 

We used the 16S rRNA gene amplicon sequencing technique to assess the bacterial community composition of the inoculum and the enrichment cultures. The bacterial community diversity in the inoculum samples was higher than in the enrichment cultures, and enrichment cultures from P2 were more diverse than enrichment cultures from P1 (Figure S1).

**Figure 2:**
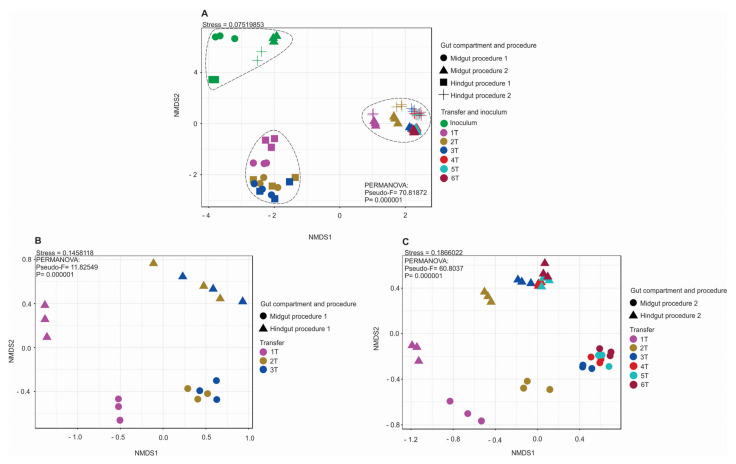
NMDS plots based on 16S rRNA gene amplicon sequences using the Bray-Curtis dissimilarity index and permutational multivariate analysis of variance (PERMANOVA) (*n* = 3). Statistics are provided as inset panels. (**A**) Inoculum, enrichment cultures of P1 and P2. (**B**) Only samples from enrichment cultures of P1 and (**C**) P2.

**Figure 3:**
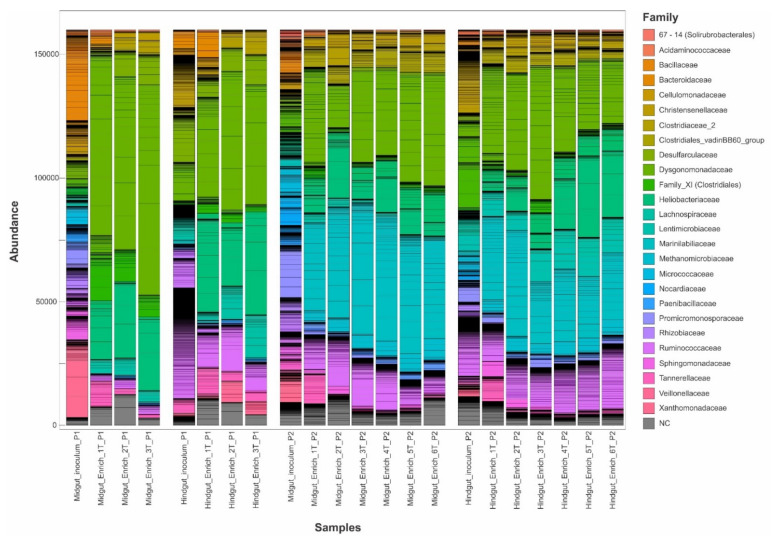
Bacterial community composition at the family level, analyzed by 16S rRNA gene amplicon sequences (*n* = 3) on day 30 of cultivation in the midgut and hindgut enrichment cultures, with three transfers in P1, six transfers for P2, and inocula from midgut and hindgut for both inoculum preparation methods. Individual genera within a color-defined family are separated by horizontal lines. The number of sequence reads was rarefied for better comparison. NC = not classified at the family level.

**Figure 4:**
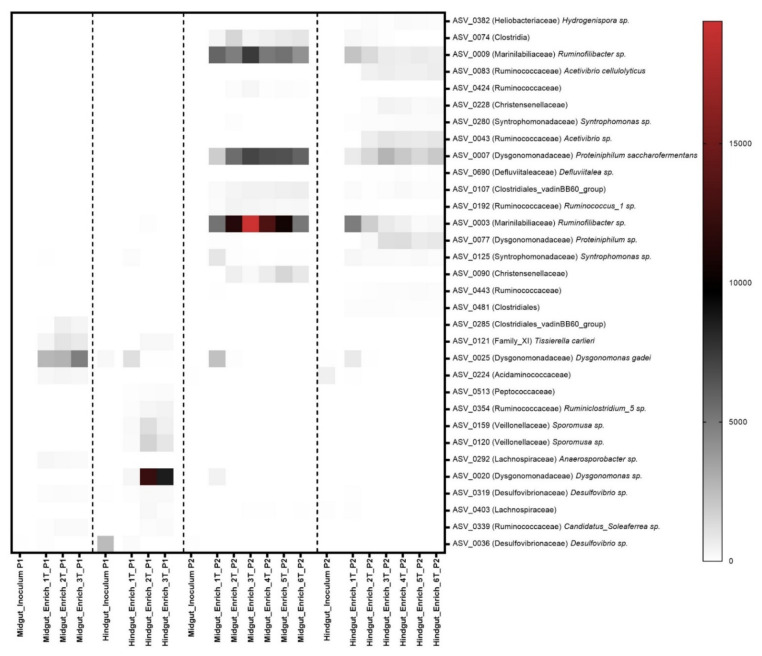
Heat map of the relative abundance of key bioindicator ASVs in enrichment cultures via P1, P2, and their inocula. The ASVs were selected using the random forest machine learning technique.

Significant differences were observed between the microbial community compositions of the inoculum and the enrichment cultures in both inoculum preparation procedures. However, there was no significant difference in bacterial community composition among transfers from the same gut compartment, except from the first transfer to the following ones (Figure 2A–C). In the current study, the enriched microbial community was stable after the second transfer (2T). One of our findings contrasts with previous studies, which have observed more dynamics of the community structures over transfers [22,86,87,88]. Lazuka and collaborators [73], during the enrichment of termite gut microbiome, reached a stable community after the fifth enrichment cycle. In particular, families such as Bacillaceae, Ruminococcaceae, and Christensenellaceae, which were highly abundant in the gut inocula in procedure 1, were outcompeted by Dysgonomonadaceae and Heliobacteriaceae during the enrichment. In procedure 2, families such as Christensenellaceae, Ruminococcaceae, and Promicromonosporaceae were outcompeted by Dysgonomonadaceae, Marinilabiliaceae, and Ruminococcaceae (Table 1). Figure 3 shows the bacterial community composition at the family level, and Figure 4 shows the key bioindicator species in the enrichment cultures and the inocula.

It is known that the digestion of organic matter rich in lignocellulose in animal guts (in vivo) is more efficient than that performed in vitro [24,89]. In addition to the microorganisms present in the gut of insect larvae, such as the *Pachnoda marginata*, the enzymes secreted by the host act synergistically to digest the content that passes through the larva’s gut. 

In general, the Dysgonomonadaceae family was present in all enrichment cultures regardless of the inoculum preparation used, though at higher abundance in enrichment cultures of P1 (Figure 3). At lower taxonomic level, the most abundant ASVs, defined as bioindicators of the Dysgonomonadaceae family, were different among the enrichment cultures (Figure 4). In the enrichment cultures obtained by P1, the *Dysgonomonas* spp. were most abundant, but the bioindicator ASVs were different in the hindgut and the midgut enrichment cultures. In the case of P2 a *Proteiniphilum* sp. was found to be a bioindicator of this family. This ASV was more abundant in the midgut than in the hindgut enrichment cultures (Figure 4). Members of the Dysgonomonadaceae family have been reported as a recurrent family in the gut environment of insects that feed on organic matter rich in lignocellulose [20,90,91,92,93,94,95,96,97] and in biogas reactors that run with lignocellulosic biomass as the main carbon source [98,99], suggesting an essential role in the cell wall degradation of plant cells [97,100]. Members of the genus *Proteiniphilum* have been described as obligate anaerobes that use proteins to produce acetic acid and propionic acid [101]. However, Wu and collaborators [98], during an investigation of the effect of intermittent microaeration in digesters, found that this genus was able to degrade lignocellulose to acetate, formate, and carbon dioxide, by anaerobic fermentation and aerobic respiration. *Dysgonomonas gadei* is a facultative anaerobic species capable of converting carbohydrates such as cellobiose, fructose, lactose, starch, sucrose, and xylose into different acids but not able to produce any gas [102].

The Ruminococcaceae family was found in all hindgut enrichment cultures and inocula regardless of the type of procedure used for inoculum preparation (Figure 3, Table 1). This shows that members of this family managed to adapt to the artificial cultivation conditions to which they were established during enrichment. During the anaerobic digestion of wheat straw bioaugmented with sheep rumen fluid, the microbial community developed a predominance of the families Lachnospiraceae and Ruminococcaceae, with a significant increase in methane production [46]. The Lachnospiraceae and Ruminococcaceae families have also been reported in studies with insects [20,63,103,104,105]. Moreover, the Lachnospiraceae family can produce butyric acid [106] and acetic acid [107].

Members of the family Marinilabiliaceae affiliated to the genus *Ruminofilibacter* were found to be bioindicators for P2, which was especially abundant in midgut enrichment cultures (Figure 4). Marinilabiliaceae (*Ruminofilibacter xylanolyticum*) were found as xylanolytic organisms with pronounced hydrolytic enzyme activity in biogas reactors operated with grass silage [108]. The Desulfovibrionaceae family was not among the most abundant groups in the procedure 1 midgut inoculum, but it was enriched in both midgut and hindgut cultures. It was not observed among the most abundant families in the inoculum or enrichment in procedure 2 (Table 1). During the enrichment of sulfate-reducing microorganisms from the gut of *P. marginata*, Dröge et al. [109] found *Desulfovibrio intestinalis* and *Desulfovibrio* strain STL1 at great abundance. The genus *Desulfovibrio* can survive for 120 h of aeration and is known to consume oxygen and hydrogen, and produce acetate [110,111,112]. In the case of low hydrogen availability, they can compete with acetogens and methanogens [109]. Egert et al. [113] found *Desulfovibrio* spp. in great abundance in the hindgut of the *M. melolontha* larva (Scarabaeidae). Ebert et al. [114] found *Desulfovibrio* spp. in great abundance in *Cephalodesmius* sp. (Scarabaeidae) and attributed the role of these microorganisms to the constant removal of oxygen in this compartment, providing an anoxic environment. *Desulfovibrio* sp. has also been widely reported in the gut of lower and higher termites [109,115] and cockroaches [116]. 

The conversion of lignin in anaerobic systems remains an enigma, as it is believed that oxygen is necessary for this process to occur. Studies have been dedicated to the chemical process of lignin conversion and determining which microorganisms are capable of carrying out this process [117,118,119]. Geib and collaborators [120] found out that lignin degradation can also occur in a different ecosystem, in the gut of wood-feeding insects, and that it is not restricted to wood-rot fungal systems. Bacteria capable of growing on lignin and on a waste product from the paper industry (black liquor) were isolated from the greenhouse insect camel cricket (*Diestrammena asynamora*) and hide beetle (*Dermestes maculatus*) [118]. Studies carried out by Scully and collaborators revealed that the Asian long-horned beetle (*Anoplophora glabripennis*), a wood-feeding insect, harbors complex microbiota capable of degrading hemicellulose, enhancing the degradation of lignin, and fermenting xylose [121,122]. On the other hand, Lemke et al. [29], investigating the microbiota of *P. ephippiata* larvae, did not observe the degradation of aromatic compounds derived from lignin. A study by Chouaia et al. [103] demonstrated a wide variation in the microbial structure of the gut of *Popillia japonica* (Scarabaeidae) depending on the larva stage development and the gut section. The most abundant families in third instar larva were Clostridiales, Ruminococcaceae, Christensenellaceae, Desulfovibrionaceae, and Rikenellaceae [103]. Sheng et al. [68] isolated 93 strains with cellulolytic activity from the hindgut of *Holotrichia parallela* (Scarabaeidae) larvae, claiming that the best strain has better cellulolytic enzymes than the ones obtained from fungi and other bacteria widely studied and that it is thermostable, increasing its biotechnological potential. The gut microbiota investigation of animals that are not conventionally studied for application in the biotechnological industry is essential for the discovery of new species capable of overcoming biotechnological barriers and contributing to the development of biotechnological processes.

### 3.3. Methanogenic Community Structure

**Figure 5:**
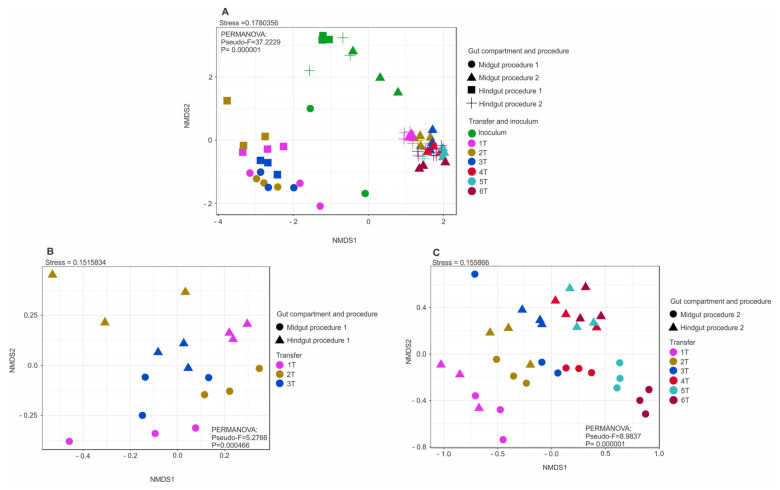
NMDS plots based on *mcrA* gene amplicon sequences using the Bray-Curtis dissimilarity index and permutational multivariate analysis of variance (PERMANOVA) (*n* = 3). Statistics are provided as inset panels. (**A**) Inocula, enrichment cultures of P1 and P2. (**B**) Only samples from enrichment cultures of P1 and (**C**) P2.

**Figure 6:**
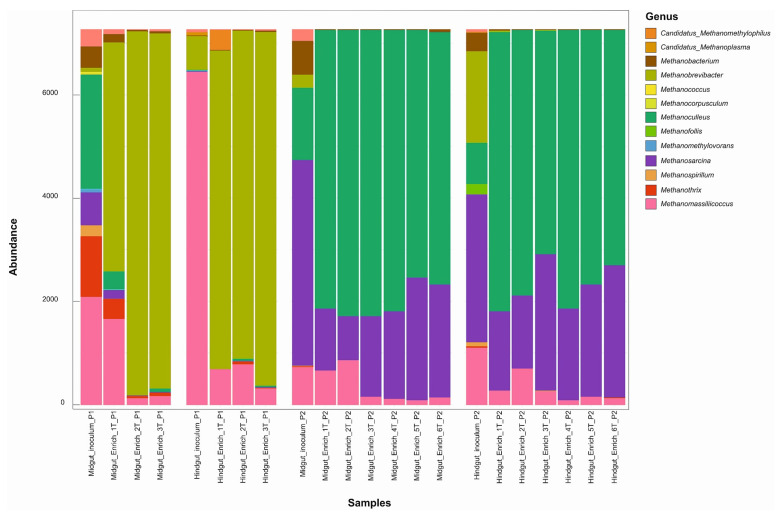
Methanogenic community composition at the genus level analyzed by *mcrA* gene amplicon sequencing at 30 days of cultivation in midgut and hindgut cultures, over three transfers (P1), over six transfers (P2), and in inocula. NC = not classified at the genus level. The bars show the average of *n* = 3. Error bars were omitted to improve the figure visibility. The number of sequence reads was rarefied for better comparison.

The methanogenic community composition in the inoculum and the enrichment cultures were assessed via amplicon sequencing of the *mcr*A gene. As expected, a decrease in the methanogenic community diversity was observed in the enrichment cultures when compared to the inoculum with both procedures (Figure S2), similar to the bacterial community diversity. Although the literature has reported low numbers or even the absence of methanogens in the midgut of insect larvae of the family Scarabaeidae, in this study, methanogens were detected by PCR and formed the midgut inoculum. The NMDS plots show that the methanogenic communities enriched with P1, P2, and the inoculum differed considerably (Figure 5). In general, transfers of P1 had a lower diversity than those derived from P2. In transfers of P1, there was a predominance of the Methanobacteriaceae family (genus *Methanobrevibacter*) in midgut and hindgut enrichment cultures (Figure 6, Figure S2). Interestingly, *Methanobrevibacter* was below the detection limit in the midgut inoculum sample in P1 but become abundant during enrichment. Egert et al. (2003) investigated the gut archaeal community of *P. ephippiata* larvae and found Crenarchaeota as the most abundant phylum in midgut, while Euryarchaeota, phylum was dominant in the hindgut with the most abundant dominant orders of *Methanobacteriales*, *Thermoplasmatales*, and *Methanosarcinales* [17]. *Methanobrevibacter* spp. were the only methanogens detected in the hindgut of *Melolontha melolontha* larvae (Coleoptera: Scarabaeidae) [113]. In goat and cow rumen enrichment cultures on wheat straw, the predominant methanogens were the strictly hydrogenotrophic Methanobacteriales and Methanomicrobiales [23]. Cazemier [89] detected methane production in enrichment cultures from *P. marginata* hindgut after feeding on beech litter and filter paper but did not investigate the methanogenic community involved in this process. *Methanothrix*, which is a strictly acetoclastic microorganism, was detected in P1 midgut inoculum, and all enrichment transfers and the second transfer (2T) of hindgut enrichment cultures as a minor community member. In P2, it was detected only in hindgut inoculum but was not established in enrichment cultures. This is the first time that strictly acetoclastic *Methanothrix* has been reported in the gut of Scarabaeidae larvae. In the enrichment cultures, the new culture conditions likely promoted the proliferation of *Methanothrix*, which was not found in abundance in the midgut of the larva. In the P2 samples, the two most abundant methanogenic families were Methanosarcinaceae (genus *Methanosarcina*) and Methanomicrobiaceae (genus *Methanoculleus*) in both midgut and hindgut cultures (Figure 6). Although hydrogenotrophic methanogenesis is dominant in the gut systems of Scarabaeidae larvae, the transient occurrence and survival of acetoclastic methanogens cannot be ruled out. The appropriate cultivation conditions may promote the growth of these minor community members. Similarly, targeted molecular investigation of a functional group at a bigger sequencing depth can also detect previously overlooked taxa.

It is known that methanogens are present in the gut of Scarabaeidae larvae. However, methane production is an unavoidable side activity of microbiomes present in animal guts [24], taking care of hydrogen and CO_2_ from biomass fermentation. Due to the larvae’s need for energy, a microbiome co-evolved in the gut system with the main function of volatile fatty acid production. VFAs, mainly acetic acid, are later absorbed by the larvae’s gut tissue [123]. However, both P1 and P2 inoculum preparation procedures enriched the enrichment cultures with methanogens. The neutral pH conditions may promote the easy establishment of this group of microorganisms throughout the enrichment process, since the ideal pH for methane production reported in the literature ranges from 6.8 to 8.5 [124,125]. This does not necessarily mean that methane production in extreme pH environments, such as soda lakes and acid rivers, does not occur [15,126,127]. Considering that the production of methane in P1 was very low and acetoclastic methanogens were absent, it might be concluded that the inoculum preparation under oxic conditions resulted in the accumulation of acids instead of biogas production. Consequently, the appropriate treatment of inocula derived from gut can be a way of steering the process toward carboxylate production.

It is known that the accumulation of VFAs can also lead to the inhibition of microorganisms responsible for acidogenesis. In the larvae’s gut, inhibition of the acidogenesis process is avoided by the constant uptake of acids by the gut tissue. Since this process does not occur in the artificial system (biogas plants and enrichment cultures of this study), the production of methane and the activity of syntrophic acetate oxidizing bacteria (SAOB) are the two ways in which the excess of acids and the consequent inhibition of the acidogenesis process are avoided. Representatives of the families Syntrophomonadaceae, Synergistaceae, and Thermoanerobacteraceae were found in the enrichment cultures of both procedures, whereas in the inoculum, they were below the detection limit. Among the three families, the most abundant was Syntrophomonadaceae. Several members of these bacterial families are described as SAOB [128,129,130,131,132]. In anaerobic systems, SAOB usually compete for acetate with acetoclastic methanogens, such as Methanosaetaceae and Methanosarcinaceae [133], that can use the acetoclastic pathway, but live in interdependency with hydrogenotrophic methanogens such as *Methanoculleus* [132] and *Methanobacterium* [134]. The oxidation products of acetate are H_2_, CO_2_, and formate, which are consumed by hydrogenotrophic partners to keep their concentration low, making the reaction thermodynamically feasible [135]. Therefore, it is assumed that the syntrophic relationship between bacteria and hydrogenotrophic methanogens might be another way of VFAs removal in the enrichment cultures. However, this role is probably less likely in the gut of *P. marginata* larva.

## 4. Conclusions

One of the main conclusions and major observations of this study is that preparing the inoculum from *P. marginata* larva gut under oxygen seemed to suppress methanogenesis but increased carboxylate production, whereas preparing the inoculum under anoxic conditions increased the methane production. Regardless of the procedure used for inoculum preparation, the diversity of the microbial communities in the enrichment cultures strongly changed compared to that observed in the larvae gut. Based on the presented results, we could infer that the inoculum preparation condition played an important role in the establishment of enrichment cultures by influencing the microbial community composition and the final metabolites. It was possible to maintain the enrichment cultures from the *Pachnoda marginata* larva gut in an artificial system to produce considerable amounts of carboxylates or methane from raw wheat straw as carbon and energy source. Further investigation of these enrichment cultures is necessary since the microbial communities have a great biotechnology potential for lignocellulosic biomass degradation. These cultures are very promising for introduction in biorefineries.

## Figures and Tables

**Table 1:** Summary of the top 5 most abundant ASVs and families in the midgut and hindgut, procedure 1 (3T), procedure 2 (6T) and inoculum samples. The relative abundance of *n* = 3 was averaged.

Strategy	Gut Compartment	Culture	ASVs	Relative Abundance (%)	Family	Relative Abundance (%)
**P1**	Midgut	Inoculum	*Pseudoxanthomonas* sp. (Xanthomonadaceae)	14	Bacillaceae	22
			*Bacillus niacini* (Bacillaceae)	5	Xanthomonadaceae	4
			*Bacillus drentensis* (Bacillaceae)	4	Promicromonosporaceae	4
			*Xylanimicrobium pachnodae* (Promicromonosporaceae)	4	Ruminococcaceae	2
			*Bacillus* sp. (Bacillaceae)	3	Enterococcaceae	1
		Enrichment	*Dysgonomonas* sp. (Dysgonomonadaceae)	32	Dysgonomonadaceae	47
			*Hydrogenispora* sp. (Heliobacteriaceae)	18	Heliobacteriaceae	17
			*Dysgonomonas* sp. (Dysgonomonadaceae)	9	Family_XI	8
			*Desulfovibrio* sp. (Desulfovibrionaceae)	6	Desulfovibrionaceae	5
			*Dysgonomonas gadei* (Dysgonomonadaceae)	6	Lachnospiraceae	4
	Hindgut	Inoculum	*Desulfovibrio* sp. (Desulfovibrionaceae)	5	Ruminococcaceae	28
			*Proteiniphilum* sp. (Dysgonomonadaceae)	5	Christensenellaceae	13
			*Desulfovibrio* sp. (Desulfovibrionaceae)	3	Desulfovibrionaceae	12
			*Bacteroides* sp. (Bacteroidaceae)	3	Dysgonomonadaceae	11
			*Parabacteroides* sp. (Tannerellaceae)	2	Lachnospiraceae	10
		Enrichment	*Dysgonomonas* sp. (Dysgonomonadaceae)	16	Dysgonomonadaceae	31
			*Hydrogenispora* sp. (Heliobacteriaceae)	12	Heliobacteriaceae	21
			*Hydrogenispora* sp. (Heliobacteriaceae)	11	Ruminococcaceae	10
			*Dysgonomonas* sp. (Dysgonomonadaceae)	9	Lachnospiraceae	7
			*Dysgonomonas* sp. (Dysgonomonadaceae)	5	Desulfovibrionaceae	10
**P2**	Midgut	Inoculum	*Xylanimicrobium pachnodae* (Promicromonosporaceae)	7	Promicromonosporaceae	12
			*Cellulosimicrobium* sp. (Promicromonosporaceae)	3	Bacillaceae	6
			*Luteimonas* sp. (Xanthomonadaceae)	3	Microbacteriaceae	4
			Lactobacillales (order) (Bacilli)	2	Enterococcaceae	4
			*Agromyces* sp. (Microbacteriaceae)	2	Xanthomonadaceae	4
		Enrichment	*Proteiniphilum* sp. (Dysgonomonadaceae)	10	Marinilabiliacea	28
			*Proteiniphilum saccharofermentans* (Dysgonomonadaceae)	8	Dysgonomonadaceae	22
			*Ruminofilibacter* sp. (Marinilabiliaceae)	6	Ruminococcaceae	10
			*Ruminofilibacter xylanolyticum* (Marinilabiliaceae)	6	Heliobacteriaceae	7
			*Proteiniphilum acetatigenes* (Dysgonomonadaceae)	6	Clostridiaceae	7
	Hindgut	Inoculum	*Eubacterium* sp. (Eubacteriaceae)	10	Christensenellaceae	16
			*Sebaldella termitidis* (Leptotrichiaceae)	4	Ruminococcaceae	15
			*Enterococcus pallens* (Enterococcaceae)	3	Eubacteriaceae	10
			Lactobacillales (order) (Bacilli)	3	Enterococcaceae	7
			*Xylanimicrobium pachnodae* (Promicromonosporaceae)	3	Lachnospiraceae	5
		Enrichment	*Hydrogenispora* sp. (Heliobacteriaceae)	16	Dysgonomonadaceae	22
			*Ruminofilibacter* sp. (Marinilabiliaceae)	6	Marinilabiliaceae	19
			NA. (Marinilabiliaceae)	5	Ruminococcaceae	12
			*Lentimicrobium* sp. (Lentimicrobiaceae)	5	Heliobacteriaceae	10
			*Lutispora* sp. (Gracilibacteraceae)	4	Lentimicrobiaceae	5

## Data Availability

Demultiplexed raw sequence data were deposited in the NCBI SRA public database under the bioproject PRJNA788342.

## Supplementary Materials

The following supporting information can be downloaded at: https://www.mdpi.com/article/10.3390/microorganisms10040761/s1, Figure S1: Alpha-diversity metrics from Inoculum, P1 and P2 samples based on 16S rRNA gene amplicon sequences, Figure S2: Alpha-diversity metrics from Inoculum, P1 and P2 samples based on *mcr*A gene amplicon sequences.
