# Supplementary material for: Enrichment of Anaerobic Microbial Communities from Midgut and Hindgut of Sun Beetle Larvae (Pachnoda marginata) on Wheat Straw: Effect of Inoculum Preparation
Source: Microorganisms. 2022 Mar 31;10(4):761. doi: 10.3390/microorganisms10040761 (PMC9024811; doi:10.3390/microorganisms10040761)
Supplement: Supplementary file 1 [file microorganisms-10-00761-s001.zip › microorganisms-1535709-supplementary.pdf]

<u>**Supplementary material:**</u>

Alpha-diversity of the bacterial communities

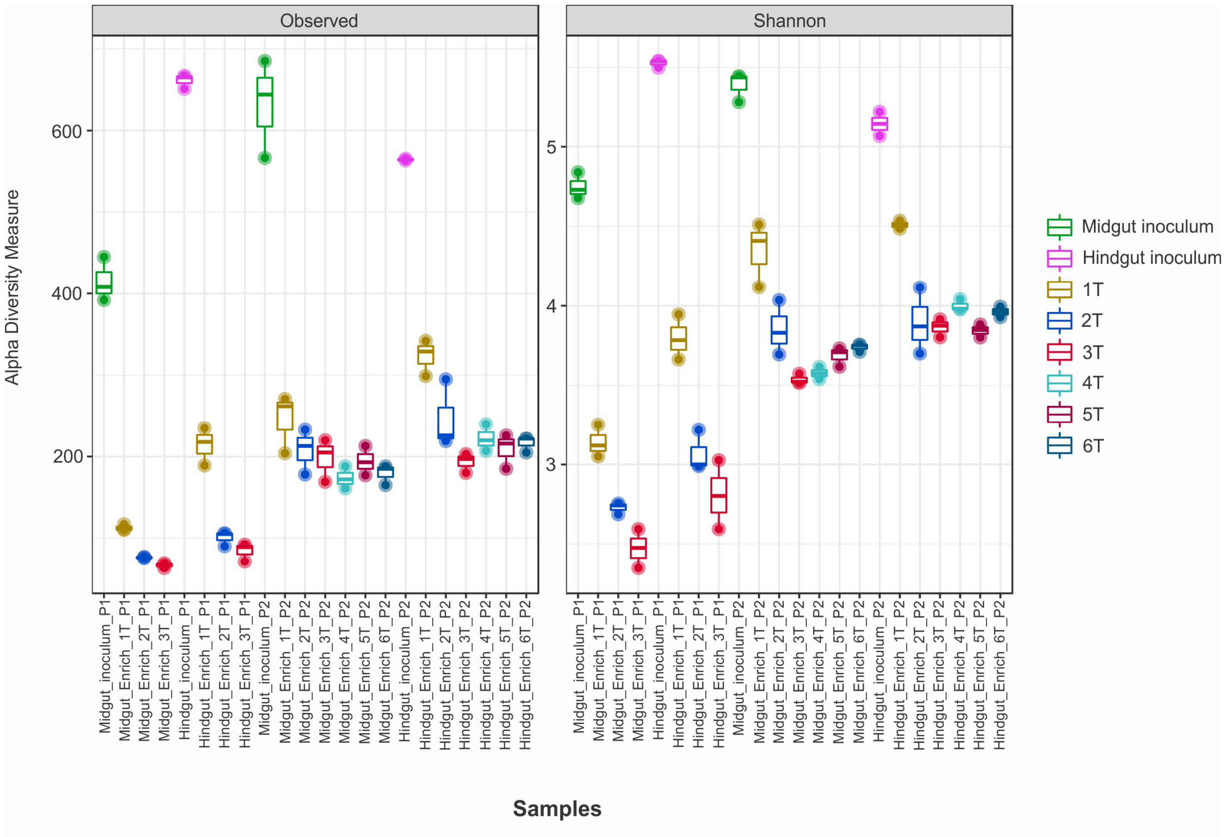

Figure S1. Alpha-diversity metrics of inocula, and enrichment culture transfer samples (1T-6T) derived from inoculum preparation procedure 1 (P1) and 2 (P2), based on 16S rRNA gene amplicon sequences (n = 3).

Alpha-diversity of methanogenic communities

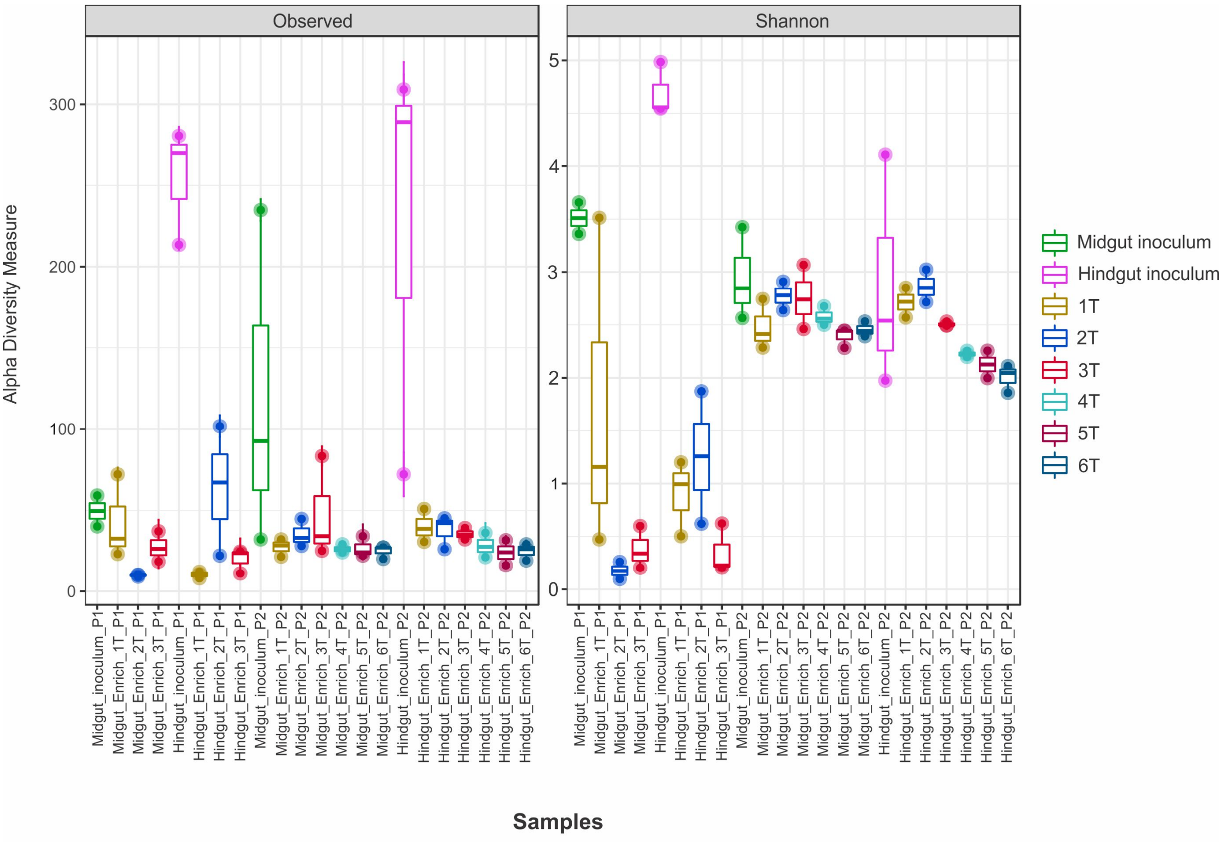

Figure S2. Alpha-diversity metrics of inocula, and enrichment culture transfer samples (1T-6T) derived from inoculum preparation procedure 1 (P1) and 2 (P2), based on *mcrA* gene amplicon sequences (n = 3).
